# How Much Do Healthcare Practitioners Know About Sarcoma?

**DOI:** 10.3390/curroncol33070394

**Published:** 2026-07-01

**Authors:** Motaz Alaqeel, Saad M. Alangari, Abdulrahman Ahmed Almebki, Abdulaziz Alderaywsh, Falwa Alarnous, Waleed Albishi, Ibrahim Alshaygy, Abdulrahman Alaseem

**Affiliations:** 1 Department of Orthopedic Surgery, College of Medicine, King Saud University, P.O. Box 2925, Riyadh 11461, Saudi Arabia; 2 College of Medicine, King Saud University, Riyadh 11461, Saudi Arabia; 3 Department of Family and Community Medicine, King Saud University Medical City, King Saud University, Riyadh 11461, Saudi Arabia; 4 College of Medicine, AlFaisal University, Riyadh 11533, Saudi Arabia

**Keywords:** soft tissue sarcoma, bone sarcoma, awareness, investigations, health care providers

## Abstract

Sarcoma awareness among healthcare practitioners in our institution was limited, prompting an evaluation of their ability to recognize early presentations and initiate appropriate work-up for soft tissue and bone sarcomas. A structured survey assessed familiarity with key clinical features, recommended investigations, and perceived contributors to diagnostic delay. Overall awareness was modest, but higher among healthcare practitioners with prior sarcoma exposure, oncology-focused training, longer experience, or heavier patient loads; female practitioners and oncology nurses also scored higher. Confidence in identifying red-flag symptoms was strongly linked to familiarity with guideline-based practice. Despite these strengths, notable gaps persisted across specialties and training levels, indicating that exposure alone is insufficient. Targeted education, improved recognition of early warning signs, and clearer referral pathways are needed to reduce diagnostic delays and support timely management of suspected sarcomas.

## Simple Summary

Sarcomas are rare tumors that are frequently overlooked, and earlier work has shown that limited clinician familiarity contributes to delays in diagnosis. In our setting, we examined how well healthcare practitioners understand the presentation and diagnostic work-up of soft tissue and bone sarcomas. Overall awareness ranged from low to moderate, with stronger knowledge seen among those who had prior exposure to sarcoma cases, worked in oncology-related roles, or had longer clinical experience. Greater awareness was also associated with higher confidence and better understanding of guideline recommendations. These results align with existing evidence that practical experience and focused education improve recognition of sarcoma. They also underscore the need for structured, case-based teaching and clearer referral pathways. Future research should explore targeted educational strategies and institutional processes that could reduce diagnostic delays and improve patient outcomes.

## 1. Introduction

Sarcomas are rare but clinically significant malignancies for which early recognition is essential, as outcomes are closely related to tumor stage and size at diagnosis. Despite established referral principles, healthcare practitioners may have limited exposure to sarcoma and may initially manage patients as having benign musculoskeletal conditions, contributing to delayed diagnosis and referral. Despite the recognized impact of delayed diagnosis and referral on sarcoma outcomes, limited data are available on healthcare practitioners’ knowledge of soft tissue and bone sarcomas, particularly regarding symptom recognition, appropriate initial investigations, and referral pathways.

Soft tissue sarcomas (STS) and bone sarcoma (BS) remain uncommon but clinically significant malignancies. STS occur at an estimated global incidence of about 3.6 per 100,000 people, underscoring their rarity and the challenge they pose to early detection [1]. Osteosarcoma, the most common primary malignant bone tumor, accounts for one-third of all cases [2]. In the United States alone, the American Cancer Society projected around 13,400 new STS cases in 2023 with an estimated 5140 deaths across both adult and pediatric population [3].

Early recognition of STS and BS is critical, as prognosis strongly depends on tumor stage and size at diagnosis, with smaller, early-stage tumors were associated with better outcomes [4]. Although advances in diagnostics and treatment have contributed to gradual improvements in survival [5], timely and accurate diagnosis remains essential for optimizing both survival and quality of life [6]. Yet, studies consistently show that clinicians often have limited experience with these rare tumors and low knowledge with existing guidelines, leading to inadequate referrals even when clinical features warrant a specialist evaluation [7]. A 2020 systematic review highlighted that many sarcoma patients are initially managed in primary or secondary care as benign musculoskeletal conditions, with clinicians frequently overlooking red-flag features such as enlarging deep soft-tissue masses, persistent bone pain at rest, or atypical radiographic findings [8]. Only a minority of patients were referred directly to sarcoma centers and specialists, reflecting gaps in knowledge of guideline recommended pathways, particularly in younger patients with limb pain or swelling [8,9]. A study showed that delays from first medical consultation to sarcoma center referral represented 49% of high-risk and 42% of low risk of total delay, and inadequate imaging had to be repeated for 51% of MRI and 18% of CT scans [10].

There remains a notable lack of published data examining the current levels of STS and BS knowledge among healthcare practitioners (HCPs). This gap limits our understanding of how clinicians recognize and manage these rare tumors. Because of this, the pattern of misdiagnosis, inappropriate initial management, and delayed referral system may imply persistent gaps in HCPs awareness of sarcoma features, diagnostic imaging algorithms, and the necessity of early referral to specialized multidisciplinary teams [8,9]. Enhancing knowledge and awareness of STS and BS is essential because higher awareness was strongly linked to earlier diagnosis, timely referral to specialized centers, and more effective therapeutic management [9]. This study aimed to address this gap by evaluating practitioners’ awareness and knowledge of sarcoma presentation and investigation, and by identifying differences across demographic and subspecialty groups to guide future educational initiatives.

**Key findings:**

1. Healthcare practitioners demonstrated variable awareness of soft tissue and bone sarcoma, with more consistent recognition of bone sarcoma presentations and greater gaps in soft tissue sarcoma red-flag recognition and diagnostic work-up.
2. The study answered the research questions by showing the current level of sarcoma knowledge, identifying differences across selected demographic and professional groups, and demonstrating that prior exposure to sarcoma cases, greater confidence, and self-reported knowledge were associated with better overall awareness.
3. In practice, clinicians should use these findings to maintain a high index of suspicion for enlarging, deep, persistent, or painful soft tissue masses and unexplained bone pain, arrange appropriate initial imaging, and refer suspected cases early to a specialized sarcoma multidisciplinary team.

## 2. Materials and Methods

### 2.1. Study Design and Participants

We surveyed HCPs who primarily see patients at a tertiary referral hospital in Riyadh, Saudi Arabia. Eligible participants included primary care physicians, orthopedic surgeons, residents, medical interns and nurses. Non-HCPs, medical students and trainee nurses were excluded. Sample size was calculated using Zα = 1.96 for a 95% confidence interval, a proportion of 20% and a margin of error of 5%. The calculated target sample size for this study was 138 participants; however, 129 healthcare practitioners were included in the final analysis. The difference between the target and final sample size was primarily due to non-response and incomplete survey submissions. Because the survey was distributed anonymously, we were unable to determine the individual reasons for non-response or incomplete participation, and we could not compare responders with non-responders. No additional participants were excluded based on demographic or professional characteristics after completion of the survey. This was considered when interpreting the findings, as non-response may have introduced selection bias if participants with greater interest in oncology or sarcoma were more likely to complete the survey.

### 2.2. Data Collection and Survey Tool

We collected the participants’ demographics including age, gender, position, subspeciality, years of experience, weekly patient volume, and prior attendance at oncology conferences. We also asked for their familiarity with sarcoma guidelines, their confidence in sarcoma management, experience in evaluation of STS and BS patients and having seen STS and BS patients. STS and BS case presentations as well as investigations were asked and participants’ knowledge were assessed on how they answered the questions correctly using a self-reported validated questionnaire by Fossum et al. [7]. The correct responses were determined a priori based on internationally recognized sarcoma guidelines and accepted diagnostic principles, including recommendations from the UK National Institute for Health and Care Excellence (NICE), the American Joint Committee on Cancer (AJCC), the Musculoskeletal Tumor Society, and other international sarcoma guidance. For soft tissue sarcoma, responses were considered correct when they reflected established red-flag features such as increasing size, deep location, size ≥ 5 cm, suspicious or indeterminate imaging findings, appropriate use of contrast-enhanced MRI, and referral to a specialized sarcoma service when clinical concern persists. For bone sarcoma, correct responses were based on warning features such as persistent or progressive localized bone pain, night/rest pain, swelling or palpable mass, abnormal radiographic findings, plain radiography as the initial investigation, MRI for further assessment when suspicion remains, and referral to a specialized bone sarcoma center before biopsy. The answer key was reviewed by two orthopedic consultants specialized in sarcoma care to confirm that each response was clinically appropriate and aligned with international diagnostic and referral recommendations.

Although the instrument was used in its original form, we conducted a full revalidation to ensure its suitability for our study population. The questionnaire underwent expert-based face and content validation by two orthopedic surgery consultants specialized in sarcoma care, who were the first and last authors of this manuscript. Each consultant had more than ten years of experience in the diagnosis and management of soft tissue and bone sarcomas, was North American trained, and had completed advanced training at an internationally recognized, top-ranked academic institution. They reviewed each item for clinical relevance, clarity, appropriateness of the case-based scenarios and response options, accuracy of the proposed correct answers, and alignment with recognized sarcoma red-flag features, diagnostic principles, and referral pathways. Their feedback was used to refine selected questions and ensure consistency with the study objectives. This process represents author-led expert face and content validation; formal psychometric validation, content validity index calculation, and test–retest reliability assessments were not performed. There was no need for translation since all participants were English speakers. The revalidated questionnaire had an internal consistency reliability of Cronbach’s alpha = 0.72. The survey was sent via email to all participants.

### 2.3. Statistical Analysis

Data were analyzed using the Statistical Package for Social Sciences (SPSS) version 28.0 statistical software (IBM-SPSS, Armonk, NY, USA). Descriptive statistics (mean, standard deviation (SD), frequencies and percentages) summarized participant characteristics and awareness scores. The total overall knowledge scores based on the correct answers were summarized as mean and SD after normality testing by the Shapiro–Wilk test was performed. Percentage of correct responses for STS and BS investigations were also reported, and Chi-square test was used to test associations across subspecialties and gender. Independent samples *t*-test was used to compare overall mean awareness scores between two groups, and one-way analysis of variance (ANOVA) compared the overall mean knowledge scores across three or more groups. Pearson correlation test was done to determine the correlation between knowledge scores and level of confidence. A *p*-value of ≤0.05 was considered statistically significant. All statistical analyses were conducted in consultation with a professional statistician, and acknowledged appropriately. Subgroup comparisons across variables such as gender, professional position, specialty, years of experience, number of patients seen per week, and prior oncology-related training were performed to explore potential patterns in the data. These analyses were exploratory in nature and were not based on a priori hypotheses. Given the exploratory intent and the descriptive focus of the study, no formal correction for multiple comparisons (e.g., Bonferroni or false discovery rate adjustments) was applied. Results from subgroup analyses should therefore be interpreted with appropriate caution.

### 2.4. Ethical Considerations

Ethical approval was obtained from the Institutional Review Board (IRB) of the College of Medicine, King Saud University, Riyadh, Saudi Arabia. Written informed consent was obtained from all participants. Confidentiality was maintained by assigning coded identifiers for analysis.

## 3. Results

We surveyed 129 healthcare providers, of which 87 (67.4%) were males and 42 (32.6%) were females. There were 37 (28.7%) consultants/fellows, 57 (44.2%) residents and interns and 35 (27.1%) nurses. The majority were in general practice (*n* = 63, 48.8%) and 68 (52.7%) had less than 5 years of practice. Out of a maximum 10, the mean rating of HCPs familiarity with sarcoma guidelines for work-up and diagnosis of STS and BS was 4.73 ± 2.3, and 4.79 ± 2.2 for their confidence in their knowledge of sarcoma management. Detailed demographics and experience with sarcoma cases are shown in Table 1.

### 3.1. Awareness of STS Cases and Investigation for STS

The proportion of correct responses to the STS case-based questions varied from 24.0% to 76.7%. Notably, 76.0% of participants incorrectly believed that a painless, superficial 2.0 cm mass requires urgent evaluation for sarcoma. In contrast, 76.7% accurately recognized that a mass accompanied by constitutional symptoms warrants urgent assessment for STS. Most respondents (*n* = 117, 90.7%) agreed that suspicious findings on X-ray or ultrasound should be followed by contrast-enhanced MRI, whereas only 59 (45.7%) identified a homogeneous T2-weighted MRI signal as a potential indicator of STS.

**Figure 1:**
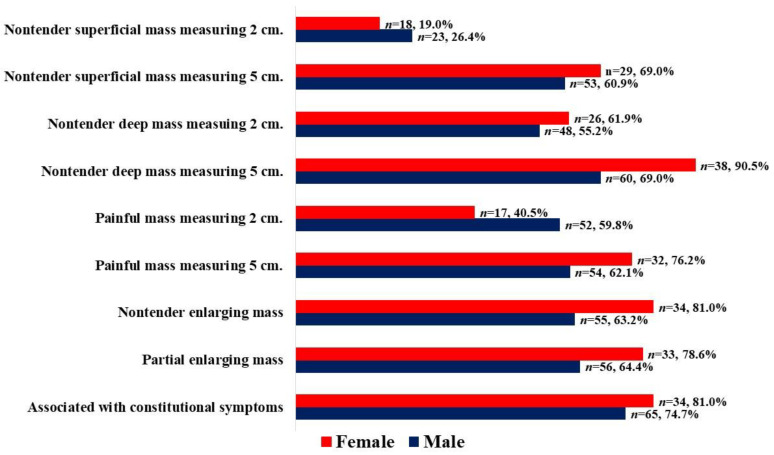
Percentage of correct responses on questions regarding soft tissue sarcoma case presentation between male and female HCPs.

Participants demonstrated variable recognition of clinical features associated with soft-tissue sarcoma, with female respondents performing better across most scenarios. Although many differences were not statistically significant, several key patterns emerged. Female respondents were significantly more likely than males to identify high-risk characteristics in two of the most clinically concerning scenarios. Recognition of a nontender deep 5 cm mass was markedly higher among females (90.5%) compared with males (69.0%; *p* = 0.007). A similar advantage was observed for nontender enlarging masses, with 81.0% of females answering correctly versus 63.2% of males (*p* = 0.041). In contrast, males outperformed females in only one scenario: the painful 2 cm mass, where 59.8% of males responded correctly compared with 40.5% of females (*p* = 0.040). Pain, however, is a less specific indicator of malignancy, which may account for the differing response patterns. For all other scenarios—including superficial masses, deep 2 cm lesions, painful enlarging masses, and masses accompanied by constitutional symptoms—accuracy was moderate and did not differ significantly between groups. Nonetheless, female respondents consistently trended toward higher recognition rates (Figure 1).

A larger proportion of consultants and fellows (59.5%) believed that a painful 2 cm mass warrants further investigation. In contrast, nurses and residents/interns were more likely to consider a painful 5 cm mass, a nontender enlarging mass, and a painful enlarging mass as indications for STS evaluation (*p* = 0.005, *p* = 0.043, and *p* = 0.031, respectively). No significant differences were observed in awareness of sarcoma presentations or appropriate investigative approaches across specialties, professional positions, years of practice, weekly patient volume, or prior attendance at oncology-focused conferences or training sessions (Table 2). Awareness of STS clinical features showed a positive—though not statistically significant—correlation with familiarity with sarcoma guidelines and confidence in sarcoma management (*r* = 0.075 and *r* = 0.016, respectively). In contrast, knowledge of appropriate STS investigations was significantly correlated with confidence in sarcoma knowledge (*r* = 0.218, *p* = 0.013).

### 3.2. Awareness of Bone Sarcoma and Its Investigation

The proportion of correct responses to the BS case-based questions ranged from 69.8% to 81.4%. Thirty-nine healthcare professionals (30.2%) did not recognize that diffuse pain persisting for one week warrants urgent evaluation for sarcoma. In contrast, 81.4% correctly identified that progressively worsening localized bone pain requires urgent workup for BS. Most respondents (*n* = 116, 89.9%) agreed that the initial step in evaluating suspected BS should be obtaining a plain X-ray.

**Figure 2:**
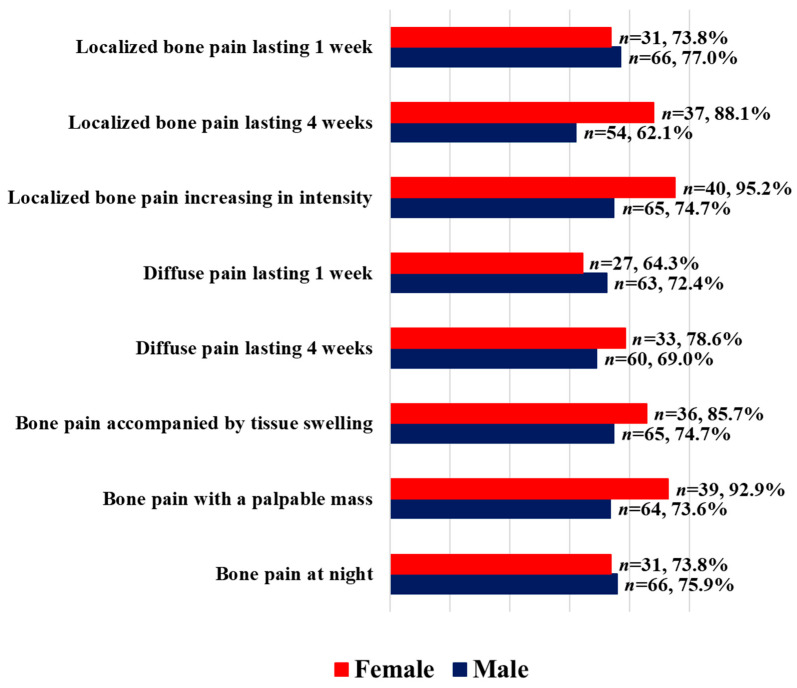
Percentage of correct answers on questions regarding bone sarcoma case presentation between male and female HCPs.

Overall, participants demonstrated a good understanding of early symptoms associated with bone sarcoma, but several notable gender-based differences emerged. Among male respondents, recognition of localized bone pain lasting one week was high (77.0%), closely mirroring the rate among females (73.8%), with no significant difference (*p* = 0.690). However, when the duration of localized pain increased to four weeks, female respondents were more likely to identify it as concerning (88.1% vs. 62.1% in males), a difference that reached statistical significance (*p* = 0.002). A similar pattern appeared for localized pain increasing in intensity, where 95.2% of females answered correctly compared with 74.7% of males (*p* = 0.005). Recognition of bone pain accompanied by a palpable mass also differed significantly, with 92.9% of females identifying it correctly versus 73.6% of males (*p* = 0.010). In contrast, both groups performed comparably on items related to diffuse pain, whether lasting one week (72.4% males vs. 64.3% females; *p* = 0.346) or four weeks (69.0% males vs. 78.6% females; *p* = 0.254). Awareness of bone pain with associated tissue swelling was also similar between genders (74.7% males vs. 85.7% females; *p* = 0.156). Recognition of night pain, another classic warning sign, showed no meaningful difference (75.9% males vs. 73.8% females; *p* = 0.800) (Figure 2).

Oncology nurses demonstrated higher accuracy in identifying BS clinical presentations—specifically localized bone pain lasting four weeks, diffuse pain of the same duration, and bone pain accompanied by a palpable mass—compared with other healthcare professionals (*p* = 0.001, *p* = 0.004, and *p* = 0.037, respectively). Consultants and fellows showed greater awareness of appropriate BS investigations, including obtaining a plain X-ray, performing a biopsy, and conducting a core Tru-cut biopsy (*p* = 0.013, *p* = 0.014, and *p* = 0.041, respectively). No significant differences were observed in awareness of BS investigative approaches across specialties, years of practice, weekly patient volume, or prior participation in oncology-focused conferences or training. Knowledge of BS clinical presentations and investigations showed positive correlations with familiarity with bone sarcoma guidelines and confidence in BS management, although these associations did not reach statistical significance (*r* = 0.406, *p* = 0.856, respectively) (Table 3).

### 3.3. Overall Awareness of STS and Bone Sarcoma

The overall mean awareness score for STS and BS was 21.53 ± 4.2 out of a maximum of 30. Healthcare professionals who had previously encountered STS or BS cases demonstrated significantly higher overall awareness compared with those without such exposure (*p* = 0.024 and *p* = 0.004, respectively). Greater confidence and higher self-reported knowledge of STS and BS were also significantly associated with overall awareness (*r* = 0.176, *p* = 0.046). No significant differences in overall awareness were observed across gender (*p* = 0.142), professional position (*p* = 0.462), specialty (*p* = 0.501), years in practice (*p* = 0.676), weekly patient volume (*p* = 0.451), attendance at oncology-related conferences (*p* = 0.341), familiarity with sarcoma guidelines (*p* = 0.568), or prior evaluation of an STS or BS case (*p* = 0.936 and *p* = 0.643, respectively). Although these differences did not reach statistical significance, higher awareness scores were observed among females, oncology nurses, those with more than 10 years of experience, individuals seeing a greater number of patients per week, and those who had attended oncology conferences.

## 4. Discussion

This study assessed healthcare practitioners’ awareness of soft tissue and bone sarcomas and identified modest, variable knowledge across clinical presentations and diagnostic pathways. Awareness was stronger for bone sarcoma than soft tissue sarcoma, while gaps remained in recognizing red-flag features and selecting appropriate investigations. Higher awareness among practitioners with prior sarcoma exposure, oncology-focused training, longer experience, greater patient volume, and higher confidence highlights the need for targeted education and clearer referral pathways to support earlier diagnosis and timely multidisciplinary care.

This study has several limitations that should be considered when interpreting the findings. First, the study relied on self-reported survey responses, which may be affected by recall bias, reporting bias, incomplete responses, or misinterpretation of individual items. Therefore, the results reflect healthcare practitioners’ stated knowledge and decision-making in case-based scenarios rather than their actual behavior in clinical practice. However, because the survey focused on practical recognition of red-flag symptoms and appropriate investigations, the findings still provide useful insight into areas where educational interventions may be needed. Second, the study was conducted at a single institution, which may limit the generalizability of the results to other healthcare settings with different referral systems, training structures, or exposure to sarcoma cases. As a result, the absolute level of awareness observed in this cohort should be interpreted in the context of our institution. However, the identification of gaps in red-flag recognition, diagnostic work-up, and referral awareness remains clinically relevant and may inform future multicenter studies and local educational initiatives. Third, although the calculated sample size was 138, only 129 participants were included in the final analysis. This shortfall may have reduced the statistical power of the study, particularly for subgroup comparisons, and may have increased the risk of Type II error. In addition, the reasons for participant exclusion or non-response were not fully characterized, which may introduce selection bias and affect generalizability. Although the final sample was close to the calculated target sample size, the study did not capture detailed reasons for participant non-response or incomplete survey participation because responses were collected anonymously. Therefore, we could not assess whether non-responders differed systematically from responders in terms of specialty, experience, prior sarcoma exposure, or interest in oncology. This may limit the generalizability of the findings and may have introduced selection bias; for example, participants with greater interest in musculoskeletal oncology may have been more likely to respond. Future studies should prospectively document participant flow, reasons for exclusion or incomplete response, and basic characteristics of non-responders when feasible. Another limitation is that, although the questionnaire was reviewed by two orthopedic consultants with experience in musculoskeletal oncology, the validation process was limited to expert-based face and content assessment. We did not conduct formal psychometric testing, calculate a content validity index, or assess test–retest reliability. Therefore, while the questionnaire was clinically reviewed for relevance and clarity, future studies should use formally validated instruments or perform comprehensive validation before wider implementation. Finally, several subgroup analyses were performed across demographic and professional variables. Because these analyses were exploratory and were not powered for hypothesis-driven comparisons, no adjustment for multiple testing was performed; therefore, the possibility of inflated Type I error cannot be excluded. Accordingly, subgroup findings should be interpreted as hypothesis-generating, while the main conclusion—that sarcoma awareness was variable and that targeted education is needed—remains supported by the overall pattern of responses.

The persistent diagnostic difficulties associated with STS and BS, along with the practical barriers clinicians encounter, underscore the need to identify realistic and effective strategies to improve early detection. Our findings show that sarcoma awareness in our institution is modest, but higher among clinicians with prior sarcoma exposure, oncology-focused training, longer experience, or heavier patient loads; female practitioners and oncology nurses also scored higher. Many clinicians remain uncertain about which early symptoms truly warrant concern. Although those with prior exposure, oncology-focused roles, or greater confidence performed better, important misconceptions persisted—such as overestimating the urgency of small superficial masses while missing more meaningful red-flag patterns in soft-tissue and bone presentations. Female practitioners and oncology nurses consistently recognized key warning signs more accurately, yet no single specialty or training level was uniformly strong. Together, these patterns highlight a clear need for more practical, targeted education and clearer referral pathways to help clinicians identify sarcomas earlier and reduce avoidable diagnostic delays.

The first purpose of this study was to evaluate the overall awareness and knowledge of healthcare practitioners regarding soft tissue sarcoma and bone sarcoma. We found low to moderate awareness of STS and BS with correct responses to STS case-based questions ranging from 24.0% to 76.0%, with variable performance across case-based questions and modest self-reported familiarity with sarcoma guidelines. This finding is consistent with prior work showing that clinicians in primary and secondary care often have limited exposure to sarcoma and low familiarity with diagnostic pathways [11,12,13,14]. Holthuis et al. showed that sarcoma patients commonly have multiple prediagnostic encounters before diagnosis, reflecting the difficulty of recognizing rare malignancies in routine practice [11]. Similarly, Fernández et al. emphasized that diagnostic delay in soft tissue sarcoma is frequently related to limited awareness, non-specific symptoms, and imperfect referral pathways [12]. Our study extends these observations by quantifying awareness among a broader institutional group of healthcare practitioners, including physicians, residents, interns, and nurses. The second purpose was to assess recognition of soft tissue sarcoma presentations and appropriate initial investigations. In our cohort, recognition of soft tissue sarcoma red flags was inconsistent, particularly when clinical features were subtle or potentially misleading, such as small, painless, superficial, or enlarging masses. This pattern aligns with the diagnostic challenge described in previous studies, where soft tissue sarcoma may initially resemble benign musculoskeletal or soft tissue conditions [11,12]. Fernández et al. noted that referral guidelines based only on clinical red flags may have limited performance because symptoms are often non-specific and may either be overlooked or over-interpreted [12]. Our finding that most participants recognized the need for contrast-enhanced MRI after suspicious initial imaging is encouraging, but incomplete recognition of MRI features suggests that education should go beyond referral triggers and include basic principles of imaging interpretation. This is supported by Iwai et al., who showed that tumor size and the relationship between a superficial mass and the fascia on MRI can help distinguish malignant from non-malignant lesions [13]. The third purpose was to evaluate awareness of bone sarcoma presentations and investigations. Compared with soft tissue sarcoma, participants showed more consistent recognition of bone sarcoma features, particularly progressive localized bone pain and the role of plain radiography as the initial investigation. This finding is broadly consistent with the previous literature emphasizing persistent, progressive, or night bone pain as important warning symptoms that should prompt imaging and further evaluation [14,15]. However, meaningful gaps remained, particularly for less classic presentations such as diffuse or short-duration pain. These gaps are clinically important because systematic reviews of bone sarcoma diagnostic delay have shown that delayed diagnosis often results from low oncologic vigilance and confusion with benign orthopedic conditions [15]. Our findings therefore reinforce the need for clinicians to maintain suspicion when bone pain is persistent, worsening, unexplained, or associated with swelling or a palpable mass. The fourth purpose was to compare awareness across demographic and professional groups and identify implications for future education. We observed higher awareness among practitioners with prior sarcoma exposure, oncology-focused training, longer experience, greater patient volume, and higher confidence in sarcoma management, while selected differences were also seen by gender and professional role. These findings are consistent with Fossum et al., who reported that practitioners with limited exposure to sarcoma had low familiarity with guidelines and frequently made decisions inconsistent with recommended work-up pathways [16]. However, because our subgroup analyses were exploratory, these differences should be interpreted as signals for targeted education rather than definitive evidence of superiority between groups. Weaver et al. also highlighted that limited experience, diagnostic uncertainty, and lack of awareness of referral protocols contribute to delayed diagnosis, supporting the need for wider dissemination of sarcoma education and referral pathways [17]. Our study extends this literature by identifying specific institutional knowledge gaps that can be addressed through focused education on red-flag symptoms, appropriate imaging, and early referral to specialized sarcoma multidisciplinary teams.

In our cohort, higher awareness of STS clinical warning signs was positively associated with familiarity with sarcoma guidelines and self-reported confidence in sarcoma management, while overall awareness remained low to moderate, with greater variability in STS recognition than BS recognition and persistent gaps in identifying red-flag presentations and appropriate work-up pathways. These findings are consistent with Fossum et al., who reported limited familiarity with bone and soft tissue sarcoma work-up guidelines among primary care practitioners and frequent management choices that were inconsistent with recommended diagnostic and referral pathways [16]. They also align with recent studies showing that sarcoma patients often experience multiple prediagnostic healthcare encounters, and that diagnostic delay is commonly related to nonspecific symptoms, limited clinician awareness, and suboptimal referral systems [17,18,19]. The relatively stronger recognition of BS in our cohort may reflect the more familiar warning pattern of persistent or progressive bone pain and the well-established role of plain radiography as an initial investigation; however, recent BS literature still shows that delayed diagnosis remains common when symptoms are mistaken for benign orthopedic conditions or when radiologic warning signs are insufficiently recognized [15,20]. Our findings therefore support prior evidence that guideline familiarity and clinical exposure are important for earlier suspicion of sarcoma and extend the literature by identifying knowledge gaps across a broader institutional sample that included consultants, fellows, residents, interns, and nurses, highlighting the need for targeted education on STS red flags, BS warning symptoms, appropriate imaging, and early referral to specialized sarcoma multidisciplinary teams.

Regarding awareness of BS and its investigation, almost one-third of our respondents failed to recognize diffuse persistent pain as warranting urgent evaluation for sarcoma, suggesting that early BS symptoms may be under-recognized when they are nonspecific or resemble benign musculoskeletal complaints. This finding is consistent with recent bone sarcoma literature showing that diagnostic delay remains common and is often related to nonspecific symptoms, low clinical suspicion, and delayed referral [15]. It also complements Hoshi et al., who found that primary physicians’ referral documents often lacked key radiological descriptors of osteosarcoma, indicating that frontline gaps may extend beyond symptom recognition to the interpretation and communication of concerning imaging findings [20]. Interestingly, Elyes et al. reported that pain can accelerate the diagnostic process by prompting earlier consultation, whereas swelling and sensory symptoms may prolong diagnostic intervals; our findings partially support this observation, as diffuse or short-duration pain may be less readily interpreted as a red flag than progressive localized pain [21]. The higher accuracy observed among oncology nurses, consultants/fellows, and practitioners exposed to sarcoma cases is also consistent with qualitative and guideline-based literature emphasizing the value of clinical exposure, specialist pathways, and multidisciplinary sarcoma care in improving timely recognition and referral [9,17,18,19,20,21,22]. Thus, our study extends existing evidence by identifying specific institutional knowledge gaps in BS recognition and work-up, supporting targeted education on persistent or progressive bone pain, suspicious radiographic features, plain radiography as the initial investigation, and early referral to a specialized bone sarcoma multidisciplinary team.

Interestingly, awareness levels did not differ substantially between healthcare professionals who reported familiarity with STS and BS guidelines and those who did not. This finding contrasts with the expected role of guideline-based education, as prior work has shown that limited familiarity with sarcoma work-up guidelines is associated with inappropriate diagnostic and referral decisions, while delayed diagnosis is often linked to limited awareness, diagnostic uncertainty, and poorly defined referral pathways [16,17]. One possible explanation is that self-reported guideline familiarity may not reflect practical, case-based application; brief or general educational exposure may be insufficient unless reinforced through repeated clinical encounters, structured case-based teaching, and clear referral algorithms [16,17]. This is particularly relevant because current STS guidance emphasizes combined red-flag assessment, including increasing size, deep location, size ≥ 5 cm, suspicious imaging features, and specialist referral, while pain alone should not be used as the primary trigger for suspicion or exclusion of sarcoma [23,24,25,26]. In our cohort, consultants and fellows appeared more willing to proceed with definitive diagnostic steps despite reassuring imaging, whereas residents, interns, and nursing staff tended to focus more on symptom evolution; this may reflect differences in clinical responsibility, exposure to complex cases, and confidence in diagnostic uncertainty. Although this differs from the Mayo Clinic survey by Fossum et al., which found no significant differences by provider type, years of experience, or prior sarcoma exposure, their cohort was limited to primary care practitioners and did not include specialists or resident physicians, which may explain the discrepancy [16]. Our findings therefore extend prior evidence by suggesting that guideline awareness alone is insufficient and that effective sarcoma education should focus on translating referral criteria into practical decision-making across different healthcare professional groups.

Delays in diagnosing and treating STS and BS have frequently been attributed to limited oncologic vigilance and the tendency to misinterpret sarcoma symptoms as more common benign musculoskeletal or orthopedic conditions [15,19]. Recent reviews have emphasized that insufficient knowledge, low awareness of red-flag features, and inconsistent use of referral pathways remain major contributors to diagnostic delay, particularly because early sarcoma symptoms may be nonspecific and easily overlooked [15,18,19]. This is consistent with our finding of low awareness among HCPs, which may influence diagnostic accuracy and the timeliness of appropriate management. Our results also align with Fossum et al., who reported limited familiarity with sarcoma work-up guidelines among primary care practitioners and frequent management decisions that were inconsistent with recommended diagnostic pathways [16]. Similarly, Holthuis et al. showed that sarcoma patients often have repeated prediagnostic healthcare encounters before referral, supporting the concern that missed or delayed suspicion in frontline settings can prolong the diagnostic pathway [18]. Current STS and BS guidelines emphasize that suspicious soft tissue masses, persistent or progressive bone pain, palpable masses, or concerning radiological findings should prompt appropriate imaging and referral to specialized sarcoma multidisciplinary teams [22,23]. Our findings extend this literature by showing that these knowledge gaps are present across a broader institutional group of healthcare professionals, reinforcing the need for targeted education on red-flag recognition, appropriate work-up, and early referral to specialized sarcoma services.

This study addresses an important gap in the existing literature by evaluating sarcoma awareness among HCPs in Saudi Arabia, where available sarcoma research has mainly focused on patient characteristics, outcomes, and public awareness rather than clinicians’ knowledge, diagnostic strategies, and referral behavior [27,28]. This gap is clinically relevant because recent Saudi data showed that patients with STS may present after prolonged symptom duration and with a substantial burden of advanced disease, underscoring the need to identify barriers to earlier recognition and referral [27]. Our findings are consistent with international literature showing that limited awareness, nonspecific early symptoms, and inconsistent referral pathways contribute to diagnostic delay in STS and BS [15,19]. By focusing on orthopedic specialists, residents, interns, and nursing staff—groups commonly involved in the initial evaluation of musculoskeletal symptoms—our study extends previous work by identifying specific educational targets within an institutional healthcare workforce. These findings support the need for targeted educational initiatives, structured institutional training, and clear referral pathways, consistent with current STS and BS guidelines and recent evidence showing that early referral to specialized sarcoma networks can improve care coordination and reduce the consequences of fragmented initial management [22,23,29]. The use of a structured, evidence-informed questionnaire also strengthened the study by ensuring a consistent approach to assessing sarcoma awareness across different professional groups.

## 5. Conclusions

This study shows that sarcoma awareness among healthcare practitioners was low to moderate, with important gaps around recognizing soft tissue sarcoma red flags, interpreting concerning presentations of bone sarcoma, and selecting appropriate diagnostic and referral pathways. Readers should interpret these findings as evidence of actionable educational and system-level gaps within routine clinical practice, rather than as definitive evidence of differences between professional subgroups. The take-home message is that improving sarcoma care requires practical, reinforced training that teaches clinicians to suspect sarcoma when encountering enlarging, deep, persistent, or painful soft tissue masses, unexplained progressive bone pain, swelling, palpable masses, or atypical imaging findings, and to initiate appropriate imaging and early referral to specialized sarcoma multidisciplinary teams. Based on these findings, institutions should implement targeted case-based teaching, red-flag checklists, clear imaging and referral algorithms, and focused training for residents, interns, nurses, and frontline clinicians who commonly evaluate musculoskeletal complaints. Future studies should use multicenter designs, validated assessment tools, and pre- and post-educational interventions to determine whether structured sarcoma education improves clinical decision-making, referral accuracy, diagnostic intervals, and, ultimately, patient outcomes.

## Figures and Tables

**Table 1:** Demographic characteristics of 129 HCPs and their experiences with sarcoma.

Characteristics	*n* (%)
Gender	
Male	87 (67.4%)
Female	42 (32.6%)
Position	
Consultants and Fellows	37 (28.7%)
Residents and Interns	57 (44.2%)
Nurses	35 (27.1%)
Specialty	
General practice	63 (48.8%)
Orthopedics	34 (26.4%)
No specialization	32 (24.8%)
Duration in practice	
<5 years	68 (52.7%)
5–10 years	28 21.7%)
>10 years	33 (25.6%)
Average number of patients seen per week	
20 and below	42 (32.6%)
21–30	39 (30.2%)
>30	48 (37.2%)
Attended conference/training in oncology	
Yes	37 (28.7%)
No	92 (71.3%)
Have evaluated STS who received a diagnosis of STS	
Yes	51 (39.5%)
No	78 (60.5%)
Have evaluated a BS patient who received a diagnosis of BS	
Yes	55 (42.6%)
No	74 (57.4%)
Have seen STS cases	
Yes	92 (71.3%)
No	37 (28.7%)
Have seen BS cases	
Yes	96 (74.4%)
No	33 (25.6%)

**Table 2:** Percentage of correct answers to questions about soft tissue sarcoma cases and investigations according to positions among 129 HCPs.

Questions on Soft Tissue Sarcoma (Case Presentation) Indicate Whether the Case Warranted Urgent Workup for Sarcoma (Yes or No)	Correct Response	*n* (%) of Correct Responses			*p* Values
		Consultants and Fellows (*n* = 37)	Residents and Interns (*n* = 57)	Nurses (*n* = 35)	
Nontender superficial mass measuring 2 cm	No	8 (21.6%)	14 (24.6%)	9 (25.7%)	0.914
Nontender superficial mass measuring 5 cm	Yes	20 (54.1%)	35 (61.4%)	27 (77.1%)	0.114
Nontender deep mass measuring 2 cm	Yes	18 (48.6%)	31 (54.4%)	25 (71.4%)	0.123
Nontender deep mass measuring 5 cm	Yes	26 (70.3%)	41 (71.9%)	31 (88.6%)	0.122
Painful mass measuring 2 cm	No	22 (59.5%)	38 (66.7%)	9 (25.7%)	<0.001
Painful mass measuring 5 cm	Yes	23 (62.2%)	32 (56.1%)	31 (88.6%)	0.005
Nontender enlarging mass	Yes	23 (62.2%)	36 (63.2%)	30 (85.7%)	0.043
Painful enlarging mass	Yes	25 (67.6%)	34 (59.6%)	30 (85.7%)	0.031
Mass associated with constitutional symptoms	Yes	29 (78.4%)	42 (73.7%)	26 (80.0%)	0.755
Questions on soft tissue sarcoma investigations. Indicate whether the statement is true or false					
The first step in STS workup is doing a lab test	False	20 (54.1%)	29 (50.9%)	16 (45.7%)	0.775
After suspicious imaging on X-ray/US we should do an MRI with contrast	True	34 (91.9%)	49 (86.0%)	34 (97.1%)	0.192
Homogenous signaling MRI T2-weighted images is an indication for STS	False	18 (48.6%)	25 (43.9%)	16 (45.7%)	0.902
Expansive and invasive growth is one of the red flags of STS that can be seen in the MRI	True	27 (73.0%)	46 (80.7%)	27 (77.1%)	0.679
Any soft tissue swelling larger than 3 cm or located in deep tissues is highly indicative of an aggressive STS	True	28 (75.7%)	37 (64.9%)	29 (82.9%)	0.154
For lesions appearing on MRI, immediate referral to a tumor center should be initiated	True	23 (62.2%)	35 (61.4%)	28 (80.0%)	0.146
Benign-appearing lesions smaller than 2 cm on MRI, always require a biopsy as initial suitable step	False	25 (67.6%)	26 (45.6%)	13 (37.1%)	0.026
Core (Tru-cut) biopsy is the method of choice for easily palpable and large tumors	True	25 (67.6%)	50 (87.7%)	27 (77.1%)	0.060

**Table 3:** Percentage of correct answers to questions about bone sarcoma cases and investigations according to positions among 129 HCPs.

Questions on Bone Sarcoma (Case Presentation) Indicate Whether the Case Warranted Urgent Workup for Sarcoma (Yes or No)	Correct Response	*n* (%) of Correct Responses			*p* Values
		Consultants and Fellows (*n* = 37)	Residents and Interns (*n* = 57)	Nurses (*n* = 35)	
Localized bone pain lasting 1 week	No	31 (83.8%)	46 (80.7%)	21 (80.0%)	0.033
Localized bone pain lasting 4 weeks	Yes	20 (54.1%)	38 (66.7%)	33 (94.3%)	0.001
Localized bone pain increasing in intensity	Yes	28 (75.7%)	44 (77.2%)	33 (94.3%)	0.070
Diffuse (more than 1 bone) pain lasting 1 week	No	31 (83.8%)	41 (71.9%)	13 (51.4%)	0.010
Diffuse (more than 1 bone) lasting 4 weeks	Yes	21 (56.8%)	40 (70.2%)	32 (91.4%)	0.004
Bone pain accompanied by tissue swelling	Yes	32 (86.5%)	37 (64.9%)	32 (91.4%)	0.004
Bone pain with a palpable mass	Yes	31 (83.3%)	40 (70.2%)	32 (91.4%)	0.037
Bone pain at night	Yes	30 (81.1%)	40 (70.2%)	27 (77.1%)	0.466
Questions on soft tissue sarcoma investigations. Indicate whether the statement is true or false					
The first step in STS workup is doing a plain X-ray	True	35 (94.6%)	54 (94.7%)	27 (77.1%)	0.013
Periosteal reaction is a classical sign related to bone sarcoma in X-ray	True	27 (73.0%)	49 (86.0%)	25 (71.4%)	0.169
Bone sarcoma is usually heterogeneously hyperintense on MRI T2 DIXON or STIR images	True	28 (75.7%)	42 (73.7%)	26 (74.3%)	0.977
Suspected bone sarcoma after an MRI scan should be referred to a specialized orthopedic center	True	34 (91.9%)	49 (86.0%)	26 (74.3%)	0.109
A biopsy can be performed in any hospital	False	27 (73.0%)	38 (66.7%)	16 (45.7%)	0.014
Core (Tru-cut) biopsy is the usual technique employed to obtain diagnostic tissue	True	24 (64.9%)	47 (82.5%)	21 (60.0%)	0.041

## Data Availability

The data presented in this study are available on request from the corresponding author.

## Abbreviations

The following abbreviations are used in this manuscript:

HCP	Health care practitioner
STS	Soft tissue sarcoma
BS	Bone sarcoma
SPSS	Statistical Package for Social Sciences
ANOVA	Analysis of variance
MRI	Magnetic resonance imaging
